# Effects of the SNAP-25 Mnll variant on hippocampal functional connectivity in children with attention deficit/hyperactivity disorder

**DOI:** 10.3389/fnhum.2023.1219189

**Published:** 2023-08-10

**Authors:** Wenxian Huang, Ahmed Ameen Fateh, Yilin Zhao, Hongwu Zeng, Binrang Yang, Diangang Fang, Linlin Zhang, Xianlei Meng, Muhammad Hassan, Feiqiu Wen

**Affiliations:** ^1^Department of Pediatric China Medical University, Shenyang, China; ^2^Healthy Care Center, Shenzhen Children’s Hospital, Shenzhen, China; ^3^Department of Radiology, Shenzhen Children’s Hospital, Shenzhen, China; ^4^Department of Pediatrics, Shenzhen Children’s Hospital, Shenzhen, China

**Keywords:** ADHD, SNAP-25, functional connectivity, hippocampus, fMRI

## Abstract

**Objectives:**

Attention-deficit/hyperactivity disorder (ADHD) is one of the most widespread and highly heritable neurodevelopmental disorders affecting children worldwide. Although synaptosomal-associated protein 25 (SNAP-25) is a possible gene hypothesized to be associated with working memory deficits in ADHD, little is known about its specific impact on the hippocampus. The goal of the current study was to determine how variations in ADHD’s SNAP-25 Mnll polymorphism (rs3746544) affect hippocampal functional connectivity (FC).

**Methods:**

A total of 88 boys between the ages of 7 and 10 years were recruited for the study, including 60 patients with ADHD and 28 healthy controls (HCs). Data from resting-state functional magnetic resonance imaging (rs-fMRI) and clinical information were acquired and assessed. Two single nucleotide polymorphisms (SNP) in the SNAP-25 gene were genotyped, according to which the study’s findings separated ADHD patients into two groups: TT homozygotes (TT = 35) and G-allele carriers (TG = 25).

**Results:**

Based on the rs-fMRI data, the FC of the right hippocampus and left frontal gyrus was evaluated using group-based comparisons. The corresponding sensitivities and specificities were assessed. Following comparisons between the patient groups, different hippocampal FCs were identified. When compared to TT patients, children with TG had a lower FC between the right precuneus and the right hippocampus, and a higher FC between the right hippocampus and the left middle frontal gyrus.

**Conclusion:**

The fundamental neurological pathways connecting the SNAP-25 Mnll polymorphism with ADHD via the FC of the hippocampus were newly revealed in this study. As a result, the hippocampal FC may further serve as an imaging biomarker for ADHD.

## 1. Introduction

One of the most common mental conditions, attention-deficit/hyperactivity disorder (ADHD), affects almost one in 20 children and adolescents globally and is characterized by fundamental symptoms of hyperactivity, impulsivity, and inattention (Bauermeister et al., 2007; Polanczyk et al., 2007; Mohammadi et al., 2021). ADHD is associated with various structural and functional abnormalities in the hippocampus, a brain region known for its importance in memory functions (Rapport et al., 2008; Kasper et al., 2012; Irwin et al., 2021).

The hippocampus plays a crucial role in spatial navigation and consolidation of information from short-term to long-term memory (Bird and Burgess, 2008; Zhong et al., 2020). Studies have reported mixed findings on hippocampal morphology in ADHD patients, with some suggesting larger volumes as a compensatory reaction to impaired temporal processing (Plessen et al., 2006), while others have related lower hippocampal volumes to more severe ADHD symptomatology (Papadopoulos et al., 2021).

As research progresses, increasing attention has been placed on the relevance of genetic factors and their relationship to ADHD, particularly through the application of neuroimaging techniques, such as resting-state functional magnetic resonance imaging (rs-fMRI), structural MRI, and diffusion tensor imaging (DTI). These studies have investigated various gene polymorphisms that could influence brain function, connectivity, and structure related to ADHD. For instance, certain dopaminergic genes, like DAT1 (SLC6A3) and DRD4, have shown significant associations with ADHD (Shaw et al., 2007; Bralten et al., 2013). rs-fMRI investigations found altered fronto-striatal FC in the presence of these gene polymorphisms, which is partly consistent with the dopamine hypothesis of ADHD (Durston et al., 2004; Posner M. I. et al., 2014). It is also worth mentioning the alterations found in striatal volumetry, where the volumes of the caudate and putamen have been tied to the DAT1 genotype (Greven et al., 2015).

Another gene of interest in ADHD neuroimaging is the brain-derived neurotrophic factor (BDNF) gene. Reductions in gray matter volume in prefrontal and limbic structures were associated with the BDNF Val66Met polymorphism, which plays a role in neuronal survival, growth, and differentiation (Gerritsen et al., 2012). Moreover, this polymorphism showed altered default mode network connectivity in individuals with ADHD, providing evidence for the involvement of BDNF in ADHD-specific alterations in FC (Lawrie, 2020; Woelfer et al., 2020).

Additionally, the catechol-O-methyltransferase (COMT) gene has been investigated in the context of ADHD. The COMT Val158Met polymorphism is associated with altered fronto-striatal connectivity (Nackley et al., 2006), and it has been linked to cognitive performance and behavioral ratings in ADHD children (Hoogman et al., 2013). Furthermore, COMT has been found to modulate the influence of the DAT1 gene on striatal volumes, suggesting a potential interaction between the two genes (Onnink et al., 2015).

Similarly, serotonin-related genes, such as the serotonin transporter (5-HTT) gene and the serotonin 2A receptor (HTR2A) gene, have also been examined, as imbalances in the serotonergic system are thought to contribute to ADHD (Oades, 2007). A study by van Rooij et al. (2015) found that both the 5-HTTLPR polymorphism of the 5-HTT gene and the T102C polymorphism of the HTR2A gene were associated with alterations in the fronto-insula-parietal network.

Furthermore, the SNAP-25 gene, encoding the SNAP-25 protein, has been shown to play a significant role in the process of synaptic vesicle fusion. This process is crucial for communication between neurons, which in turn is critical for memory and learning (Jahn and Scheller, 2006). The protein’s role is particularly highlighted in the hippocampus, a brain region known for its importance in the formation of new memories. SNAP-25’s involvement in synaptic plasticity- a key mechanism in learning and memory- has been reported in several studies. For instance, a study by Osen-Sand et al. (1996) discovered that SNAP-25 is a critical component of the synaptic vesicle fusion machinery that enables the fast, calcium-triggered release of neurotransmitters. Furthermore, SNAP-25 levels were found to be elevated in the hippocampus of rats during learning tasks, suggesting their involvement in memory consolidation (Hardingham et al., 2010). The protein’s impact on axonal growth could also indirectly affect memory and learning processes. A study by Martinez-Arca et al. (2003) showed that SNAP-25 regulates axonal elongation and specification, suggesting its role in the establishment of neural networks, which form the physical substrate of memory. Moreover, SNAP-25’s association with ADHD has been linked to its role in cognitive functions, including memory. ADHD is characterized by attention deficits and hyperactivity but often involves impaired memory function as well. Therefore, the association between SNAP-25 and ADHD could provide an indirect line of evidence for the role of SNAP-25 in memory and learning (Gizer et al., 2009). A study by Gosso et al. (2006) further strengthens the case for the involvement of SNAP-25 in human intelligence. By studying two different Dutch cohorts, the researchers found that variations in the SNAP-25 gene were associated with differences in human intelligence. Since intelligence is closely tied to learning and memory, this provides additional evidence for SNAP-25’s role in these processes.

While there have been some studies investigating hippocampus-related functional connectivity (FC) alterations in ADHD (Posner J. et al., 2014; Kowalczyk et al., 2022), the relationship between hippocampal FC and ADHD symptomatology requires further exploration. Our study aims to expand existing knowledge with a focus on hippocampal FC alterations and their connection to deficiencies in working memory. Furthermore, we consider the potential effects of the SNAP-25 Mnll variant on hippocampal FC in children with ADHD, building on previous findings that demonstrated significant relationships between rs3746544 polymorphisms and brain connectivity, as well as working memory (Wang et al., 2018; Yang et al., 2022).

Consequently, the SNAP-25 gene has been shown to play a role in ADHD. In this study, we further investigate its impact by categorizing ADHD patients into subtypes based on their SNAP-25 genotypes. We hypothesize that these genotypic subtypes might exhibit different patterns of functional connectivity, thus providing a more nuanced understanding of ADHD.

We expect our findings to provide additional insights into the underlying neurobiological mechanisms linking SNAP-25 gene variations with ADHD symptomatology and working memory deficits. This deeper understanding may not only enhance the accuracy of ADHD diagnosis but also contribute to the development of personalized intervention strategies. For instance, if the effects of the SNAP-25 Mnll variant on hippocampal FC are found to be significant, targeted therapies could be designed to address these changes and improve working memory in children with ADHD.

## 2. Materials and methods

### 2.1. Participants

This study recruited 60 children with ADHD aged between 7 and 10 years from Shenzhen Children’s Hospital through a carefully planned collaboration with local schools and pediatric clinics. Our primary goal was to investigate the relationship between hippocampal functional connectivity (FC) alterations and ADHD symptomatology, as well as assess the impact of these alterations on working memory performance in children with ADHD. Prior to enrollment, clinicians at the Shenzhen Children’s Hospital identified potential participants and informed their parents about the research study. All participants and their parents were interviewed by experienced clinicians to confirm or exclude a diagnosis of ADHD or any other psychiatric disorder using a clinical interview and the Schedule for Affective Disorders and Schizophrenia for School-Age Children–present and lifetime version (K-SADS-PL) (Kaufman et al., 1997), based on the DSM-V criteria (Association and Others, 2013).

Children with ADHD were required to meet the following inclusion criteria: (1) 7–10 years old, (2) educated in private or public schools, and (3) diagnosed with ADHD. Healthy control subjects had the same age and education requirements as ADHD subjects. The exclusion criteria for both groups included a history of head injury with loss of consciousness, severe physical disease or neurological abnormalities, drug or substance misuse, full-scale IQ measured by Wechsler Intelligence Scale for Chinese Children-IV (WISC-IV-Chinese) below 70, prescription medications for ADHD or other medical conditions used over the long term, and comorbid conduct disorder or Oppositional Defiant Disorder (ODD).

To better understand the relationship between ADHD symptoms and participant characteristics, we assessed ADHD symptomatology using the Conners’ Parent Rating Scale (Conners et al., 2011), which encompassed various factors such as delinquent behaviors, learning problems, psychosomatic disorders, hyperactivity, anxiety, and impulsivity.

The MRI scans were only performed on participants who were right-handed dominant, had no visible abnormalities on their MRI images, and did not have a history of claustrophobia. ADHD participants presented with six or more inattentive symptoms as well as six or more hyperactive/impulsive symptoms, and in subsequent statistical analysis, the summing severity scores of each symptom were used as indicators of symptom severity.

As discussed in section “2.3. Genotyping for the detection of SNAP-25 MnII variants” below, we subdivided these ADHD subjects into two subgroups based on their SNAP-25 rs3746544 genotypes, to study the potential correlation between certain genotypes and FC alterations in ADHD symptomatology. The breakdown was as follows:

1. TT homozygotes: 35 participants
2. G-allele carriers (TG): 25 participants

The healthy control group did not undergo genotyping, as our study was focused exclusively around ADHD symptomatology and the potential connection to specific genotypes.

All children and their parents were informed about the purpose and procedures of the study. Children gave their assent to participate, and their parents provided written informed consent on their behalf. The Shenzhen Children’s Hospital Medical Ethics Committee approved this study. All methods were performed in accordance with relevant guidelines and regulations.

### 2.2. Assessments of ADHD symptoms, cognitive function, and clinical outcomes

Attention-deficit/hyperactivity disorder patients, along with TT homozygotes, G-allele carriers, and HCs, underwent an exhaustive set of cognitive and behavioral evaluations. Parents of the participating children completed the Conners’ Parent Rating Scale (Conners et al., 2011). This comprehensive questionnaire assesses a variety of behavioral and cognitive concerns, capturing key factors such as learning problems, hyperactivity, anxiety, impulsivity, and delinquent behavior.

Cognitive function was evaluated using the Wechsler Intelligence Scale for Children, Fourth Edition- Chinese version (WISC-IV-Chinese) (Yang P. et al., 2013), a globally accepted intelligence test for children aged 6 to 16 years. The WISC-IV-Chinese, administered by trained professionals, provides a Full-Scale Intelligence Quotient (FSIQ) along with several other indices including the Verbal Comprehension Index (VCI), the Perceptual Reasoning Index (PRI), the Working Memory Index (WMI), and the Processing Speed Index (PSI).

These evaluations are crucial to understanding the cognitive and behavioral profiles of our participants. In this study, we employed the original summary scores for each index from the WISC-IV-Chinese and each factor from the Conners’ Parent Rating Scale. Our comprehensive assessments of ADHD symptoms and cognitive functions provide us with a rich dataset that can be correlated with the observed FC alterations in our study. Such correlations may elucidate the clinical implications of the FC alterations we identified and may provide further insights into the symptomatology and cognitive profile associated with ADHD.

### 2.3. Genotyping for the detection of SNAP-25 MnII variants

Following the manufacturer’s guidelines, peripheral venous blood samples were collected from the participants, and SNAP-25 rs3746544 genotyping was carried out using the Flexi Gene DNA Kit (QIAGEN, Germany). Rs3746544 had a forward primer of 5′ TTCTCCTCCAAATGCTGTCG 3′ and reverse primer of 5′ CCACCGAGGAGAAAATG 3′. EX-Taq polymerase and GC buffer (Takara, Dalian, China) were used in a thermocycler to perform the polymerase chain reaction (PCR) amplification (Biometra, Germany). A denaturing cycle at 94°C for 2 min was followed by 30 cycles of 94°C for 30 s, 52°C for 30 s, 72°C for 45 s, and finally an extension step at 72°C for 8 min, in the PCR technique. The TT homozygote group had 35 members (TT group = 35), whereas the TG group had 25 G-allele carriers (TG group = 25).

We decided to concentrate on the TT homozygotes and the TG G-allele carriers. The distribution of these genotypes in our sample is approximately balanced (TT group = 35, TG group = 25). This distribution was not designed to reflect population prevalence or vulnerability to ADHD. Instead, it aimed to provide a balanced dataset for comparing functional connectivity patterns.

### 2.4. Resting-state fMRI data acquisition

The Radiology Department of Shenzhen Children’s Hospital in Shenzhen, China, used a 3.0-T system scanner (Siemens Magnetom Skyra) to collect rs-fMRI data from each participant. The following parameters were used in the echo-planar imaging (EPI) process to acquire the rs-fMRI data: repetition time (TR) = 2000∼ms; echo time = 30∼ms; flip angle = 90°; matrix size = 64 \times 64; 32 axial slices; field of view = 24 \times 24 cm^2^; slice thickness = 3 mm; no gap. Structure 3D-MPRAGE: T1 Repetition Time [TR, ms] = 2300 ms, Echo Time [TE, ms] = 2.26; Number of Averages = 1.0, Slice Thickness = 1.0 mm, Field of View (FOV) = 256 mm.

### 2.5. Data pre-processing

The DPARSF (v5.1) toolkit (Yan et al., 2016) was used to preprocess the data using SPM12.^1^ The image preprocessing in the analysis as follow: the first 10 volumes were dropped because of the instability of the initial magnetic resonance imaging data and the participants’ adaptation to the experimental apparatus. First, the slice time corrected, remaining 230 volumes were realigned to account for the head movement. The head motion criterion of translation <3 mm or rotation <3° in any direction was used to retain all patients with ADHD. Subsequently, the data were normalized and resampled into 3 mm× 3 mm × 3 mm voxels. The time course of each voxel was regressed to remove unwanted factors such as the global signal, white matter signal, cerebrospinal fluid signal, and Friston-24 parameters of head motion. To lessen the impact of low-frequency drift and high-frequency physiological noise, the data were linearly detrended, filtered at 0.01–0.08 Hz, and smoothed with a 6 mm full-width-at-half-maximum Gaussian kernel. The calculation of frame-wise displacement (FD) across the time point for each participant was then performed to evaluate head motion. The bad time point and its 1-back and 2-forward volumes were finally estimated by cubic spline interpolation using scrubbing methods (Power et al., 2012) at an FD threshold of 0.5 mm.

### 2.6. Head of motion

Jenkinson et al. (2002) relative root-mean-square method was used to eliminate the mean FD produced during the scanning procedure. The mean FD (Jenkinson) was calculated to assess voxel-wise motion differences between the three groups. The mean FD did not differ significantly between the [HC (0.05 ± 0.02), TT homozygotes (0.05 ± 0.04) and G-allele carriers (0.05 ± 0.1)] groups (*P* < 0.6).

### 2.7. Hippocampus FC statistical analysis

**FIGURE 1:**
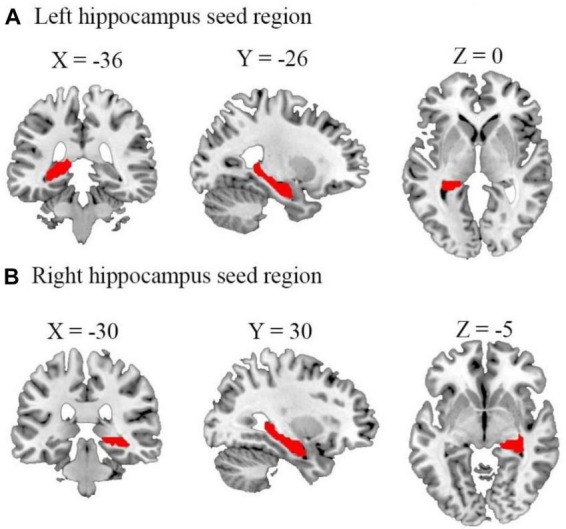
Representation of the seed regions for left and right hippocampus. The figure shows axial, coronal, and sagittal views of the brain with seed regions for **(A)** left and **(B)** right hippocampus highlighted. These regions were selected as the regions of interest for our functional connectivity analyses. Coordinates are presented in the Montreal neurological institute (MNI) standard space (left hippocampus: *x* = –36, *y* = –26, *z* = 0; right hippocampus: *x* = –30, *y* = 30, *z* = –5).

To compare the FC maps between the three groups (TT, TG, and HC), one-way ANOVA was performed. Age and FD were regressed as covariates. The basic threshold used for the data provided here was voxel-wise *p* < 0.001, cluster-level *p* < 0.05, and GRF-adjusted. Then, by averaging the Z-scores of each peak coordinate, we identified the functional connectivity signals that revealed distinct variations across the three groups. Brain regions that had undergone numerous compression corrections were subjected to *post hoc* analysis of two-tailed ANOVA tests to ascertain the direction of FC change between the three. Statistical significance was defined as *p* < 0.05/4 (Bonferroni’s corrected). Using the Montreal Neurological Institute (MNI) atlas, a standardized and internationally recognized spatial framework, we identified the exact location and boundaries of the hippocampus. We aligned our subjects’ brain images to this atlas, effectively superimposing the predefined hippocampal region onto each subject’s brain image. We then visually confirmed the alignment and made necessary adjustments to ensure an accurate fit. This rigorous process ensured the consistency of our hippocampal ROI across all subjects. Figure 1 illustrates the process of alignment and confirmation. Table 2 provides a summary of the different brain areas.

### 2.8. Partial correlation analysis

The FC values that significantly deviated from the baseline were extracted, and partial correlation analysis was used to examine the relationship between the altered FC (FC values for FSIQ and the mean value of the right precuneus) and ADHD symptom severity, working memory scores, and Conners’ scale scores. This was done while controlling for nuisance covariates such as age, grade, and mean FD. An uncorrected *p* < 0.05 threshold was used as the statistical significance criterion.

It is important to note that IQ was not included as a covariate in this analysis. While IQ is often used as a covariate in many neurodevelopmental studies, there are compelling reasons to avoid such an approach. As argued by Dennis et al. (2009), IQ scores are highly dynamic, reflecting an individual’s overall functional outcomes shaped by a myriad of factors, including genetics, biological status, cognitive capabilities, educational attainment, and personal experiences. Therefore, employing IQ as a covariate could potentially result in an “overcorrection” of our data, yielding skewed or counterintuitive results. Given these methodological considerations, we opted not to control for IQ in our analysis.

### 2.9. ROC analysis

The feasibility of employing z-variance as a diagnostic biomarker for differentiating patients with ADHD from HCs was evaluated using a support vector machine (SVM) built into the LIBSVM library [LIbrary for Support Vector Machines (Chang and Lin, 2011)]. Based on the data from the primary sample, ROIs were specifically determined from group analysis.

Our ADHD patient groups utilized in the ROC analysis comprised two genotypes: 35 TT homozygotes and 25 G-allele carriers (TG). For the SVM classification, these ADHD patient groups were split into two: one for training the classifier (*n* = 30) and another for testing (*n* = 30). These groups were randomly selected from our total pool of 60 ADHD patients and were statistically comparable in terms of demographic information and clinical characteristics. As detailed in Table 1, which provides a breakdown of demographic and clinical characteristics for the training and testing groups, we found no significant differences between these two ADHD groups (*p* > 0.05 for all variables), confirming the comparability of these groups for SVM classification.

**TABLE 1:** Demographic and clinical characteristics of ADHD training and testing sets.

Variables	Training set (*n* = 30)	Testing set (*n* = 30)	Statistics	*p*-value
Age, mean ± SD	8.4 ± 0.81	8.6 ± 0.79	*t* = 0.8	0.43
Sex (male)	M	M	χ^2^ = 0.0	1.00
Grade, mean ± SD	2.9 ± 0.51	2.7 ± 0.55	*t* = 1.2	0.23
IQ scores, mean ± SD	85 ± 7.05	86 ± 8.61	*t* = 0.6	0.55
WMI, mean ± SD	89.5 ± 9.31	88.2 ± 9.75	*t* = 0.5	0.61
VCI, mean ± SD	82.8 ± 8.61	83.5 ± 8.41	*t* = 0.3	0.76
PRI, mean ± SD	94.9 ± 11.50	96.2 ± 11.75	*t* = 0.4	0.69
PSI, mean ± SD	93.5 ± 11.40	92.9 ± 11.80	*t* = 0.2	0.84
Delinquent behaviors, mean ± SD	1.13 ± 0.51	1.15 ± 0.49	*t* = 0.2	0.83
Learning problem, mean ± SD	1.95 ± 0.57	1.98 ± 0.59	*t* = 0.2	0.84
Psychosomatic disorder, mean ± SD	0.27 ± 0.31	0.26 ± 0.30	*t* = 0.1	0.91
Hyperactivity, mean ± SD	1.62 ± 0.68	1.61 ± 0.65	*t* = 0.1	0.92
Anxiety, mean ± SD	0.67 ± 0.56	0.68 ± 0.55	*t* = 0.1	0.91
Impulsivity, mean ± SD	1.59 ± 0.49	1.60 ± 0.48	*t* = 0.1	0.92

ADHD, Attention deficit hyperactivity disorder; HC, Healthy control; SD, Standard deviation; WMI, Working Memory Index; VCI, Verbal Comprehension Index; PRI, Perceptual Reasoning Index; PSI, Processing Speed Index. The statistics column refers to *t*-values obtained by independent samples *t*-test (for continuous variables) or χ^2^-values obtained by chi-square test (for categorical variables).

**TABLE 2:** Characteristics of demographics of the three genotypic groups.

Variables	HC (*n* = 28)	TT homozygotes (*n* = 35)	G-allele carriers (*n* = 25)	Statistics	*p*-value
Age, mean ± SD	8.9 ± 0.97	8.31 ± 0.79	8.84 ± 0.71	*F* = 0.8	0.01^1^
Sex (male)	M	M	M		
Grade, mean ± SD	3.03 ± 0.83	2.8 ± 0.45	2.6 ± 0.57	*F* = 0.7	0.02^1^
IQ scores, mean ± SD	90 ± 8.40	84 ± 6.93	87 ± 9.79	*F* = 1.9	0.04^1^
**WISC-IV-Chinese**					
Working memory index (WMI), mean ± SD	97.64 ± 9.95	90.11 ± 8.70	85.02 ± 9.87	*F* = 24.80	0.05^1^
Verbal comprehension index (VCI)	103.61 ± 8.82	82.11 ± 7.91	83.25 ± 11.93	*F* = 48.130	0.048^1^
Perceptual reasoning index (PRI)	109.36 ± 11.61	96.78 ± 11.60	93.85 ± 10.76	*F* = 13.737	0.07^1^
Processing speed index (PSI)	103.64 ± 10.67	93.72 ± 11.24	92.35 ± 14.36	*F* = 7.263	0.02^1^
**Corners’ parent rating scale**					
Delinquent behaviors	0.63 ± 0.21	1.08 ± 0.55	1.18 ± 0.47	*F* = 8.322	0.05^1^
Learning problem	0.74 ± 0.25	2.03 ± 0.54	1.86 ± 0.63	*F* = 43.050	0.032^1^
Psychosomatic disorder	0.15 ± 0.16	0.27 ± 0.31	0.26 ± 0.33	*F* = 1.200	0.307^1^
Hyperactivity	0.61 ± 0.31	1.59 ± 0.70	1.64 ± 0.64	*F* = 20.153	0.050^1^
Anxiety	0.45 ± 0.21	0.66 ± 0.55	0.68 ± 0.58	*F* = 1.444	0.243^1^
Impulsivity	0.44 ± 0.22	1.61 ± 0.51	1.47 ± 0.46	*F* = 18.660	0.02^1^

HC, healthy control; ADHD, attention deficit hyperactivity; SD, standard deviation.

^1^One-way analysis of variance. F-values were obtained by 1-way ANOVA.

Although the genotypes were not differentiating factors in the classifier training, they represent significant data that contribute to a comprehensive understanding of ADHD symptomatology and FC alterations.

For feature selection, the average ROI value for each participant in the primary sample was retrieved and utilized as a feature. Prior to SVM classification, feature data were normalized within each ROI using z-score normalization (mean subtraction followed by standard deviation scaling). This normalization process ensured that features had equal weighting and did not introduce bias to the classification performance.

To train the classifier, the normalized averaged ROI values were used. In order to distinguish patients with ADHD from HCs in the independent sample, the averaged z-variance values of the same ROIs were obtained for each participant, normalized, and then input into the classifier as features.

The statistical significance of the classification performance [area under the curve (AUC) of the receiver operating characteristic curves (ROC)] was determined using a non-parametric permutation test. The actual group labels (ADHD and HC) were randomly shuffled in each permutation test trial, and the same classification process was used to determine the classification accuracy score based on the shuffled dataset. This process was repeated 5,000 times to assess the level of statistical significance and obtain the *p*-value.

## 3. Results

### 3.1. Demographic characteristics and clinical variables and clinical variables

Three TT homozygotes patients were excluded from further analyses due to excessive head motion. The final cohort contained 35 TT homozygotes, 25 G-allele carriers, and 28 HC (Table 2). No significant differences are found in term of age, gender, education level, handedness, mean FD, and the variance of FD among the three groups (Table 2). A total of 35 TT homozygotes, 25 G-allele carriers, and 28 HC groups differed significantly in clinical variables (Table 2). Patient demographic and descriptive statistics are listed in Table 2. Based on the psychometric evaluations presented in Table 2, there are some noticeable differences between the TT homozygotes and the G-allele carriers. TT homozygotes demonstrated lower scores in all WISC-IV-Chinese indices compared to HC, and G-allele carriers showed even lower scores in the Working Memory Index. In terms of behavioral assessments from Conners’ Parent Rating Scale, both TT homozygotes and G-allele carriers exhibited higher levels of delinquent behaviors, learning problems, hyperactivity, and impulsivity compared to HC, while no significant differences were found in the manifestation of psychosomatic disorder and anxiety.

### 3.2. Main effect diagnosis FC between the three groups TT, TG, and HCs in the right/left

**FIGURE 2:**
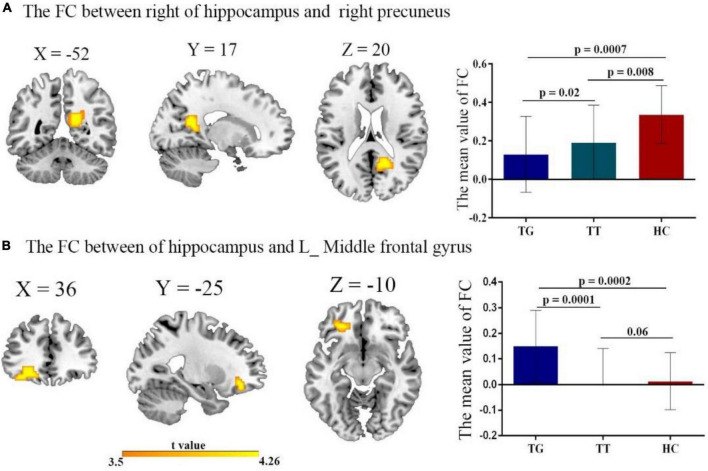
The FC of the left/right hippocampus with brain regions after comparing all three groups TT, TG, and HC with *t*-value of the FC connectivity variance; **(A)** right hippocampus with right precuneus. Decreased FC in both TT group, and TG group, compared with HC group. **(B)** Left hippocampus with left middle frontal gyrus. Reduced FC in both TT and TG group.

The 1-way ANOVA results indicated significant differences in FC variability among the three groups (TT, TG, and HCs) for the hippocampus seed regions (Figure 2 and Table 3; voxel *P* < 0.001, cluster *P* < 0.05/2, controlling for age, grade level, and the mean FD). Significant differences were observed between the three groups in both the left and right hippocampus seeds. Notably, significant differences in FC were found between the right hippocampus seed and right precuneus (Figure 2A), as well as between the left hippocampus seed and left middle frontal gyrus (Figure 2B).

**TABLE 3:** Brain clusters showing a significant effect in the FC with right and left of the hippocampus.

Seed region	Group differences	Cluster size	Z-score	M N I	TT homozygotes (*n* = 35)	G-allele carriers (*n* = 25)	HC (*n* = 28)
				X Y Z	M ± SD	M ± SD	M ± SD
Right hippocampus	Right precuneus	60	−5.09	16−56.18	0.19 ± 0.20	0.33 ± 0.19	0.33 ± 0.14
Left hippocampus	Left middle frontal gyrus	59	3.3	−27 ± 38.13	−0.04 ± 0.14	0.15 ± 0.10	0.01 ± 0.10

SD, standard deviation; M, mean value.

To further examine these differences, we conducted *post hoc* two-sample *t*-tests for each significantly different region (Figures 2A, B) among the two groups. Table 3 presents the significant effects on FC for both the right and left hippocampus seeds. A comparison of the TT and HC groups revealed a decreased FC between the right hippocampal seed and precuneus, whereas the TG group exhibited a decreased FC in the right hippocampal seed when compared to the HC group (Figure 2A). Additionally, the TT group demonstrated a decrease in FC between the left middle frontal gyrus and the left hippocampus seed when compared with the HC group (Figure 2B). In contrast, the TG group displayed increased FC between the left hippocampus seed and left middle frontal gyrus compared with the HC group as shown in Figure 2B.

### 3.3. Correlation between FC and clinical variables

**FIGURE 3:**
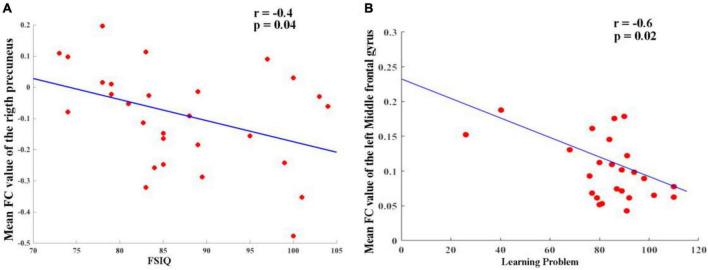
**(A)** Altered FC between right hippocampus and right precuneus in TG group and it was negatively correlated (*r* = –0.40, *p* = 0.04). **(B)** Altered FC between left hippocampus and left middle frontal gyrus in TT group and it was negatively correlated (*r* = –0.6, *p* = 0.02).

As shown in Figure 3, the partial correlation analysis, controlling for age, showed that altered FC between the right hippocampus and right precuneus in the TG group was negatively correlated with IQ scores (*r* = −0.40, *p* = 0.04) (Figure 3A). It also showed that altered FC between left hippocampus and left middle frontal gyrus in TT group was negatively correlated (*r* = −0.6, *p* = 0.02) (Figure 3B). No significant correlations were found between other changes in the FC and other regions when accounting for the control covariates.

### 3.4. ROC analysis

**FIGURE 4:**
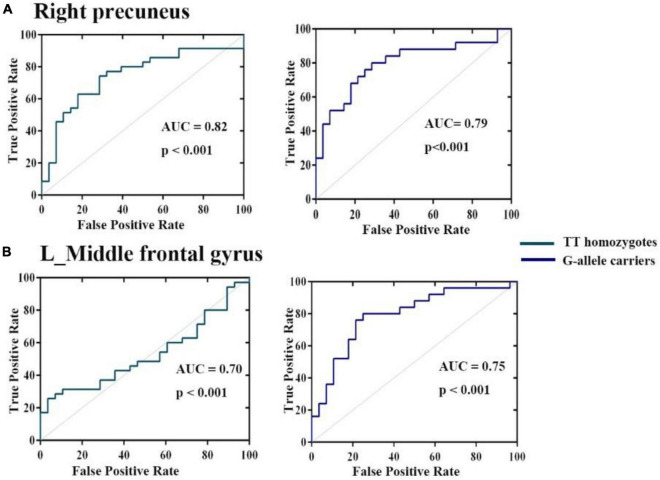
Operating characteristic (ROC) curves for discrimination between three groups for mean variance of FC **(A)** right hippocampus **(B)** left middle frontal gyrus. The AUCs of these ROIs were higher than those expected by chance (Bonferroni’s corrected).

Receiver operating characteristic analysis confirmed that HCs and patients with ADHD can be distinguished based on FC between the right hippocampus, right precuneus, and left middle frontal gyrus. As shown in Figure 4, the AUCs of these connection variability were significantly higher than those expected by chance (Bonferroni’s correction). We obtained AUCs proportions between 0.7 and 0.82, which introduces an acceptable discrimination.

## 4. Discussion

In this study, we built upon our previous research (Wang et al., 2018; Yang et al., 2022) to investigate the hippocampal involvement in working memory deficits in children with ADHD, considering the role of SNAP-25 MnII variants. Using a novel diagnostic subtyping based on SNAP-25 MnII variant genotyping, we identified distinct ADHD patient groups: ADHD-TT and ADHD-TG. Our results highlighted altered hippocampal FC among the three groups (TT, TG, and HCs), with both TG and TT patients displaying decreased FC between the right hippocampus and precuneus compared to HCs. Furthermore, we found that TG patients exhibited greater FC between the left hippocampus and left middle frontal gyrus than both TT patients and HCs. In our discussion, we delve into these findings and their implications on cognitive functioning and IQ-scores in childhood ADHD.

### 4.1. Bilateral heterogeneous hippocampus FC profiles and their effects on cognitive functioning in children with ADHD

Memory and spatial navigation are both attributed to the hippocampus. The left and right hippocampi have often been regarded as functionally equal in rats, and this bilateral brain region has been widely researched. Recently, the molecular and morphological properties of the neural connections in the brain hemisphere have been found to exhibit unanticipated asymmetries (Shipton et al., 2014). Similarly, Gu et al. (2022) found that only aberrant left hippocampal connectivity was related to cognitive function in patients with relapsing-remitting multiple sclerosis (RRMS). Our study supports two major interrelated findings: (1) we observed a bilateral distinct hippocampal FC profile with heterogeneous connections to different brain regions, and (2) these FCs may impact cognitive functioning in children with ADHD, taking into account the obtained IQ scores.

We found altered FC between the right hippocampus and the (right) precuneus in both the TT and TG patient groups compared to the healthy control group (HCs). Disrupted FC between the left hippocampus and the (left) middle frontal gyrus was observed in patient groups. The precuneus, involved in a wide range of complex tasks such as memory, information integration, mental imagery, and affective reactions to pain, has been extensively reported in correlation with hippocampal FC, especially regarding mild cognitive impairment and Alzheimer’s disease (Kim et al., 2013; Xue et al., 2019).

Ren et al. (2018) highlighted the reciprocal connectivity between the hippocampus and precuneus, emphasizing their relevance to metacognition in settings similar to daily life. Our study added to this evidence by suggesting a negative correlation between changes in FC between the right hippocampus and the right precuneus in the TG group and reduced IQ scores (*r* = −0.40, *p* = 0.04). In addition to the previously mentioned findings, our study has further identified a significant negative correlation between altered FC in the left hippocampus and the left middle frontal gyrus in the TT group (*r* = −0.6, *p* = 0.02). This adds another dimension to our understanding of how ADHD may impact brain connectivity and cognitive abilities. It is becoming increasingly clear that genetic variations in ADHD patients play a pivotal role in their brain function, contributing to the symptomatic heterogeneity observed in this disorder. The results underscore the importance of considering the interplay of genetic factors and functional connectivity when examining the complex manifestations of ADHD and its associated cognitive deficits.

The left middle frontal gyrus is crucial for literacy development. In Chinese reading, the left middle frontal gyrus serves as a specialized hub region that connects ventral and dorsal pathways (Guo et al., 2022). In dyslexic Chinese readers, structural and functional impairments in the left middle frontal gyrus contrast with the left temporoparietal regions in alphabetic languages (Yang Y. et al., 2013). This might explain why we observed disrupted FC between the hippocampus and the left middle frontal gyrus in our Chinese ADHD patients. Future research should further explore the implications of these FC alterations on other aspects of cognitive and behavioral functioning, as well as their potential as targets for intervention. Our findings suggest that different genetic groups within ADHD may require tailored treatment approaches taking into account their specific patterns of brain connectivity.

Regarding the cognitive implications of these FC alterations, the co-occurrence of lower IQ scores and academic achievement deficits and learning problems in ADHD children is well-established. We observed decreased FC values between the left hippocampus and the left middle frontal gyrus in the TT group compared to the TG group, although not statistically significant. Similar findings were reported by Siok et al. (2009), who discussed how dyslexic reading in Chinese individuals is represented by phonological impairments assessed through the weak activity of the left middle frontal gyrus in a rhyme-judgment task.

These altered hippocampus-precuneus-middle frontal gyrus connections may clarify the compromised attention-related encoding and retrieval processes leading to cognitive deficits in ADHD (Ortega et al., 2020). A thesis by Roya (2016) hypothesized that ADHD patients possess decreased resting-state activity in pathways through the hippocampus because of reduced volume and executive functioning frequency. However, that study revealed no abnormal connectivity in the hippocampus. These contradicting results highlight the need for further hippocampus research in ADHD populations.

### 4.2. SNAP-25 as a sensitive marker of disrupted patterns of connectivity in ADHD

Our first main finding was that FC alterations within the hippocampal FC in Chinese children with ADHD are genetically driven through observations of SNAP-25 variants. If replicated, this result indicates that SNAP-25 is a sensitive marker of disrupted connectivity patterns in ADHD. Although the relationship between SNAP-25 and altered structure or connectivity patterns has been repeatedly reported in patients with bipolar disorder (Houenou et al., 2017), autism (Braida et al., 2015), schizophrenia, and major depressive disorder (Najera et al., 2019), it is remarkable that only a few studies have investigated the implication of SNAP-25 in connectivity alterations in ADHD (Wang et al., 2018; Yang et al., 2022). One possible reason for the relationship between SNAP-25 and these psychiatric disorders is that SNAP-25 has a genetic basis that is linked to some symptoms that co-occur and are common among these disorders. For the SNAP-25 itself, although changes in neurotransmitter release have been suggested as potential causative processes, the mechanisms by which abnormalities in SNAP-25 may contribute to certain mental illnesses, including ADHD, remain unclear. Intriguingly, in line with our findings, Braida et al. (2015), suggested that the SNP rs363050 has a regulatory region based on analysis of transcriptional activity, which resulted in a reduction in protein expression. This reduction in protein expression affected the teenage mice, whose levels of SNAP-25 were reduced. Accordingly, they exhibited hyperactivity, cognitive and social dysfunction, and irregular EEG signals with numerous spikes.

On the other hand, Houenou et al. (2017) demonstrated that compared to non-risk carriers (of a promoter variant in SNAP25, rs6039769 at-risk allele), male risk carriers had a larger amygdala and increased FC between the amygdala and ventromedial prefrontal cortex. Therefore, this study supports the finding that this allelic variation of SNAP25 has a functional effect on modulating the development and plasticity of the prefrontal-limbic network, which may increase vulnerability to both early onset bipolar disorder and schizophrenia. Based on these studies, to better understand the abnormalities of the hippocampal FC in ADHD patients, we also need to analyze more about the genetic variations of SNAP-25 and ADHD.

The presynaptic plasma membrane protein SNAP-25 is abundantly and selectively expressed in nerve cells (Söllner et al., 1993). Considering the functions of SNAP-25, it is likely that any variation in this protein, which is mainly and distinctively encountered in axons and nerve terminals (Wang et al., 2014), can influence vulnerability to ADHD by affecting neurotransmitter release and the formation of neural circuits throughout the central nervous system (CNS). Based on its physiological significance in the docking and fusion of synaptic vesicles in presynaptic neurons as well as in axonal growth and synaptic plasticity, SNAP-25 is a potential candidate gene for ADHD. The C allele of rs1051312 is increasingly being transmitted in Canada cases (Barr et al., 2000). Using a transmission disequilibrium test (TDT), Brophy et al. (2002) demonstrated favored transmission of the T allele of rs1051312 among Irish ADHD patients. Chinese (Gao et al., 2009) and Colombian (Gálvez et al., 2014) populations showed a significant association between rs3746544 (1065T > G) and ADHD in case-control studies, but the Irish (Brophy et al., 2002), Indian (Sarkar et al., 2012), Canadian (Barr et al., 2000), US Caucasian (Feng et al., 2005), and UK Caucasian (Mill et al., 2004) groups showed no such association. In two separate samples of families with ADHD, 12 SNPs were examined by Feng et al. (2005). They discovered significant over-transmission of the rs66039806-C, rs362549-A, rs362987-A, and rs362998-C alleles in a Canadian sample but not in a southern California sample. These alleles were located in introns 2, 4, and 6. When they used quantitative analysis to assess a Canadian population for the behavioral ADHD subtypes of inattention and hyperactivity, they discovered relationships between both categories and SNAP-25. However, several studies (Ilott et al., 2010) have found no evidence to support a link between these polymorphisms and ADHD.

However, consistent with our findings, SNAP-25 was found to encode a protein that is crucial for synaptic vesicle fusion and neurotransmitter release. Furthermore, recent studies have indicated that SNAP-25 is involved in learning and memory, two processes essential for human cognition and intelligence (Noor and Zahid, 2017). Single nucleotide polymorphisms (SNPs) in genes associated with cognitive function have been reported in patients with ADHD. Barr et al. (2000) were the first to detect the Mnll polymorphism (rs3746544), a relatively often researched SNP found in the 3′-untranslated region (3′-UTR), which is linked to ADHD. Early in Chen et al. (2008) indicated that SNPs in the 3′-UTR constitute a crucial microRNA-binding site and may alter binding sites while demolishing the operating site or generating another illegitimate site (Chen et al., 2008), which could impact the expression of the SNAP-25 gene and may ultimately increase the susceptibility for the progression of ADHD (Németh et al., 2013; Ye et al., 2016).

Although our study does not provide direct clinical applications of SNAP-25 genotyping in ADHD, it suggests the possibility of subtype-specific FC patterns. This might mean that TT homozygotes and G-allele carriers could have differences in ADHD presentation, which could potentially inform future diagnostic and treatment strategies. Further research is necessary to verify these findings and explore their clinical implications in more detail.

### 4.3. Limitations

We acknowledge that our study has several limitations despite providing evidence from many levels. First, the sample size of the fMRI study was rather small; therefore, caution should be exercised when interpreting data. Whole-brain structural and connectivity analyses could potentially be performed using independent samples with a larger sample size. Second, replication of our findings is problematic because of the small number of accessible brain samples. The investigation of SNAP-25 expression levels in various regions depending on the genotype would be of significant interest because of the critical role of the hippocampus in ADHD and the regions that have been demonstrated to be functionally connected. However, changes in the SNAP-25 level and its SNARE complex binding partners have frequently been observed in mouse models and humans with psychiatric illnesses, highlighting the significance of this complex’s control. Moreover, replication from multiple polymorphisms in subjects among genetically different ethnic groups can also reveal significant findings. Even though the analyses took age into account, further evaluations of individuals who had reached adulthood could provide more light on developmental features. Another limitation is that although we examined the relationship between the hippocampus and cognitive deficits, our study did not take into account a more comprehensive psychological assessment of the cognitive parameters associated with ADHD. Therefore, future research can include more features, particularly those related to children’s performance at school or at home. Other patient-specific factors were also reported to limit our study, such as male preponderance, existence of comorbidities, and heterogeneity in clinical presentation, which are typically prevalent in ADHD studies. Therefore, to obtain more accurate results, further research should use more complex patient groups, in which both genetic and symptomatology-based subtypes are considered.

## 5. Conclusion

A major obstacle in understanding neuropsychiatric illnesses is the functional characterization of disease-associated variants, which will open an avenue for the creation of personalized therapies. According to a growing body of research, the SNARE complex, and more specifically, the SNAP-25 protein, may play a role in mental diseases. Here, as a first step, we were able to corroborate the association of one of the SNAP-25 variants with ADHD by providing modest evidence for the *Mnl*I marker of SNAP-25 with ADHD. Based on genotyping for the detection of SNAP-25 MnII variants, we were able to obtain a new subtyping of patient groups (TT and TG) for further application in functional imaging analysis. In other words, instead of adopting the typical symptomatology-based ADHD subtypes, we use genetics-based subtypes, which we believe provides more accurate findings, especially for heritable and genetic disorders such as ADHD. For our FC analysis, we investigated the role of the hippocampal FC in childhood ADHD and its possible association with cognitive impairment. In view of the influence of SNAP-25 on disease processes, an additional thorough integration of genetics-based investigations with neuroimaging is essential to pave the way for larger, more varied, and in-depth genome-wide association studies.

## Data availability statement

The datasets generated and/or analyzed during the current study are not publicly available because of Chinese Ethics Committee regulations but are available from the corresponding author (FW, fwen62@126.com) on reasonable request.

## Ethics statement

The studies involving human participants were reviewed and approved by the Medical Ethics Committee of the Shenzhen Children’s Hospital. Written informed consent to participate in this study was provided by the participants’ legal guardian/next of kin.

## Author contributions

FW, HZ, BY, and AF designed the study. WH, YZ, DF, and LZ collected the data and organized the clinical information. FW, AF, BY, and HZ reviewed the methods. AF, WH, and DF analyzed the data. WH and AF wrote the manuscript. All authors discussed, approved, and proofread the results and the final manuscript.

## Footnotes

1
https://www.fil.ion.ucl.ac.uk/spm/software/spm12/
